# Nano urea effects on *Pleurotus ostreatus* nutritional value depending on the dose and timing of application

**DOI:** 10.1038/s41598-021-85191-9

**Published:** 2021-03-10

**Authors:** Youssef N. Sassine, Layla Naim, Zeina El Sebaaly, Sami Abou Fayssal, Mohammed A. Alsanad, Milena H. Yordanova

**Affiliations:** 1 Department of Plant Production, Faculty of Agriculture, Lebanese University, Beirut, Lebanon; 2 Department of Agricultural Biotechnology, College of Agricultural and Food Sciences, King Faisal University, P.O. Box 400, Al Ahsa, 31982 Saudi Arabia; 3 Department of Agronomy, Faculty of Agronomy, University of Forestry, 10 Kliment Ohridski Blvd, 1797 Sofia, Bulgaria; 4 Department of Environment and Agricultural Natural Resources, College of Agricultural and Food Sciences, King Faisal University, P.O. Box 400, Al Ahsa, 31982 Saudi Arabia

**Keywords:** Biological techniques, Biotechnology

## Abstract

The work investigated the effect of Lithovit-Urea50 on the composition of *Pleurotus ostreatus* (Jacq. Ex Fr.) P. Kumm. (1871) cultivated on spent oyster substrate mixed with wheat straw (1:1, w/w mixture). The product was applied in different doses (C_1_: 3 g kg^−1^ and C_2_: 5 g kg^−1^) at three distinct timings (t_1_: at spawning, t_2_: after first harvest, t_3_: at spawning and after first harvest). Protein and fiber contents increased respectively by 0.64 and 0.2% in C_1_t_1_ and by 0.46 and 0.8% in C_2_t_2_ compared to control (C_0_t_0_). Total carbohydrates increased by 0.48–3.76%. Sucrose and glucose contents decreased in the majority of treatments, while fructose increased in C_2_t_1_ (by 0.045%). Essential amino acids were the highest in C_1_t_1_, wherein respective improvement of 0.31, 0.10, 0.05, 0.21, 0.18, and 0.09% compared to C_0_t_0_. Similarly, C_1_t_1_ was superior in non-essential amino acids. Potassium, sodium, calcium, iron, and copper contents decreased in all treatments, with minor exceptions, zinc decreased in C_1_t_1_ and C_2_t_1_, while nickel and lead increased in all treatments. Conclusively, despite important ameliorations in the mushroom nutritional value, mostly in C_1_t_1_, the product should be further tested in lower doses (< 3 g kg^−1^) to counteract its effect on heavy metal bioaccumulation.

## Introduction

*Pleurotus ostreatus*, commonly known as oyster mushroom, can grow in a wide range of temperatures using available lignocellulosic materials^1^. It is the second most commercially produced mushroom worldwide^2,3^. Oyster mushrooms are treasured for their low calorie, high protein, zinc, chitin, fiber, and vitamins (C, D, and B-complex) contents^4^, as well as for their amino acid composition^5^. Determining the nutritive content of oyster mushrooms could enhance their therapeutic value^6^. It is known that mushroom composition and nutritional value are affected largely by the type and nutrient composition of the substrate^7–9^. Effectively, the nutritional value of oyster mushrooms varied when they were cultivated on different substrates ^10–12^.

In mushroom producing areas, spent mushroom substrate (SMS) accumulates in huge quantities in collection centers of mushroom producing areas^13^, as 5–6 kg of SMS is the result of the production of 1 kg of fresh mushroom^14^. This leftover biomass derived from mushroom production^15^ gained special interest in early scientific reports for the cultivation of oyster mushroom. In fact, it is an abundant, cheap and easily available source of substrate, and is highly nutritive^16^. It contains good amounts of cellulose, hemicellulose, calcium, magnesium, potassium, proteins, and carbohydrates^17–19^. Oyster mushroom can degrade the lignocellulosic compounds of SMS, by producing hemicellulases, cellulases, and ligninases enzymes^20^. The bioconversion of this waste material by the mushroom has been optimized through the addition of nutritional additives^21^. Effectively supplementation of the spent substrate with commercial nutritional supplements^22,23^, or other protein-rich additives^24^ has not only caused a remarkable increase in *P. ostreatus* yields, but it also ameliorated the mushroom nutritional value^25,26^.

Overall, important aspects have to be considered during supplementation, including the type of nutrients required^27^, the choice of supplement, and the most correct timing and methods for it to be applied^28^. For instance, urea has been used as an organic source of nitrogen to improve oyster production, with a varying effect depending on the timing^29^ or dose of application^30^ to the growing substrate. Besides, it ameliorated the mushroom nutritional value by increasing both their proteins and β-glucans contents^31^. However, the use of nano-urea may be practically and economically more advantageous in mushroom farming compared to the conventional urea. The higher nitrogen content in the latter (46% N compared to 21% N in nano-urea) poses increasing risks of contamination of the substrate by competing fungi. On the other hand, nano-urea shows a slower releasing pattern of nitrogen into the substrate, providing a more efficient utilization of the applied nutrients, by applying a lesser amount of fertilizer^32^.

Furthermore, knowing that the use of nano-urea as nutritional supplement to the spent oyster substrate (SOS) gave promising results regarding the productivity of oyster mushroom^33^, the present work aimed to discuss the effect of nano-urea addition in different timing and doses on the nutritional value and amino acids composition of *P. ostreatus*.

## Material and methods

### Experimental design

*Pleurotus ostreatus* mushroom was cultivated on a half-half mixture of wheat-straw based spent oyster substrate (SOS) (obtained after one growing cycle of oyster mushroom at a local mushroom farm “Gourmet”; Mehrin, Jbeil, Byblos, Lebanon) and wheat straw (1:1, w/w mixture). The spent oyster substrate was initially sun-dried for 1 week, then shopped and mixed with wheat straw. The mixture was pasteurized at 60–65 °C, for 8 h, using hot water. Then, the mixture was cooled down to spawning temperature (25 °C). Spawning was performed at a 5% rate, corresponding to 50 g kg^−1^ of substrate, using grain spawn of M2175 strain (Mycelia Company, Deinze, Belgium)^33^.

To evaluate the effect of supplementation on the mushroom nutritional value, Lithovit-Urea50 was tested in two separate doses: C_1_, 3 g kg^−1^, C_2_, 5 g kg^−1^), and three timings of application (t_1_, at spawning; t_2_, after first harvest; t_3_, at spawning and after first harvest). Therefore, six experimental treatments (C_1_t_1_, C_1_t_2_, C_1_t_3_, C_2_t_1_, C_2_t_2_ and C_2_t_3_) were arranged in a full factorial design with two factors (dose and timing of application) and ten replicates (bags) per treatment. The different treatments were compared to a non-treated substrate or control (C_0_t_0_). Inoculated substrates were filled into perforated transparent polyethylene bags of 60 cm length and 40 cm width.

Lithovit-Urea50 is a nitrogen fertilizer, suitable for use in organic farming (pursuant to Regulation (EC) No. 834/2007-European Community), sourced from Tribodyn AG Company, Northelm, Germany, and having the following composition: 33.0% calcium carbonate (CaCO_3_), 21.0% total nitrogen (N), 18.5% calcium oxide (CaO), 6.5% silicon oxide (SiO_2_), 1.2% magnesium oxide (MgO), 0.5% iron (Fe), and 0.01% manganese (Mn). The product was initially created by a process of tribodynamic activation and micronization of dolomite^34^.

### Environmental conditions

Inoculated bags were put in a cropping chamber in dark conditions at 23–25 °C until the end of the mycelia run stage. The cropping chamber was well-sealed and equipped with climate control facilities. During the mycelia run stage, the room was continuously moistened to keep relative humidity levels in the range of 80–90% (using Hotsale 7 L/h Ultrasonic Mist Maker). It was kept close to keep the CO_2_ levels high for an adequate development of mycelium. At complete mycelia run (14 days after incubation), pin head formation was triggered by ventilation, reduction of the CO_2_ levels to a range of 900–2300 ppm, lighting (200 lx light source), and reduction of the room temperature to around 15 °C. A high relative humidity (88–90%) was maintained during the fruiting stage. Continuous monitoring of the room temperature and relative humidity was performed using a humidity/temperature meter Lutron HT-3007SD.

### Analysis of substrate properties

Properties of the initial substrate (Table 1) were analyzed as follows: moisture content (using Moisture Analyzer, Sartorius Instrument, Model MA37, Göttingen, Germany), organic matter (via loss of ignition method at 430 °C over 24 h, C/N ratio using CHN Analyzer with automatic sampler, Carlo-Erba elemental analyzer, Model 1106, Milan, Italy), and pH using a pH meter (UltraBasic-UB10; Denver Instrument, Bohemia, New York, USA). The determination of the total protein content followed the AOAC official methods of analysis^35^, using Micro-Kjeldahl method (N × 6.25). Total carbohydrates were determined using Anthrone method^36^. Furthermore, cellulose, hemicellulose, and lignin content were analyzed on dry samples (initial and residual substrate) using ANKOM technology method, filter bag technique, following AOAC official methods of analysis^37–39^.

**Table 1:** Properties of the basic substrates (dw: dry weight).

Components	WS + SOS (1:1, w/w mixture)	WS	SOS
Organic matter (%dw)	82.8	92.7	82.6
Total protein (%dw)	7.50	6.5	8.0
Total carbohydrates (%dw)	30.54	35.2	28.2
Cellulose (%dw)	35.95	41.05	31.51
Hemicellulose (%dw)	13.14	21.38	10.6
Lignin (%dw)	6.59	7.27	6.05
Moisture (%)	85.6	80.6	83.5
C/N ratio	43:1	50:1	40:1
pH (1:5, w/v)	5.2	5.8	5.0

### Analysis of mushroom composition

Around one kilogram of complete (pileus and stipe) and mature fruiting bodies of each experimental treatment were needed to perform the different tests of composition. The different tests were performed on fresh samples, and then results were provided in percentage dry weight by conversion. Total protein content of samples was estimated by the macro-Kjeldahl method (N × 4.38)^40^. Determination of total carbohydrates content followed the Anthrone method^36^. Fat content was determined by extracting a known weight of powdered sample with ethyl ether, using a Soxhlet apparatus. The percentage of fiber was calculated, based on AOAC official method 962.09^41^ general method, after determining the ash content in mushrooms (2 g of mushrooms put in a crucible and set in a muffle furnace at 500 °C for 12 h, then the furnace removed, cooled in a desiccator and weighed). Thereafter, 0.5 g of dried sample were extracted using acetone in a 50 ml centrifuge tube, the extract containing fat was discarded, and 50 ml of H_2_SO_4_ (1.25%) were added, the mixture was boiled at 100 °C for 15 min, then centrifuged at 1000 rpm, decanted and the solution discarded. This was followed by the addition of 50 ml NaOH (1.25%), boiling at 100 °C for 15 min, centrifugation at 1000 rpm, decantation, discarding again the solution, adding 25 ml H_2_SO_4_ (1.25%), decanting solution, adding 50 ml H_2_O, filtering the solution on a pre-weighed filter paper using Buchner, washing with 2 H_2_O portions 50 ml each then using 25 ml ethanol portion. Therefore, the residue was dried for 2 h at 130 °C in oven; the remaining residue represented fiber and ash contents. The percentage of fiber was then calculated (Eq. 1).

1 $$\%fiber=\frac{\mathrm{weight }\left(\mathrm{paper}+\mathrm{residue}\right)-\mathrm{ weight }(\mathrm{paper})}{\mathrm{weight }\left(\mathrm{sample}\right)-\%\mathrm{crude ash}}$$

The analysis of amino acid composition followed ISO standards (13903:2005)^42^. Free amino acids were extracted with hydrochloric acid. Co-extracted nitrogenous macromolecules were precipitated with sulfosalicylic acid and removed by filtration. The filtered solution was adjusted to a pH of 2.2. Amino acids were then separated by ion exchange chromatography and determined by reaction with ninhydrin with photometric detection at 570 nm.

For the measurements of ions (Ca, K, Mn, Fe, Na and Mg), 2.8 ml of HNO_3_ (65%) were added to 5–6 g of samples, digested at 150 °C for 1 h, then filtrated with 100 ml of distilled water. The filtrate was then subjected to Inductively Coupled Plasma Mass Spectrometry (ICP-MS). Phosphorus content was determined by spectrophotometric method, wherein phosphorus reacts with molybdic acid to form phosphomolybdate complex, which was then reduced with amino naphthol sulfonic acid to complex molybdenum blue that was measured spectrophotometrically^43^.

Zinc, copper, nickel and lead were determined using Atomic Absorption Spectrophotometer (AAS) (Perkin Elmer, Model Analyst 400, Waltham, Massachusetts, USA); mushroom samples were digested using a mixture of HNO_3_, H_2_SO_4_, and H_2_O_2_ (4:1:1) (12 ml per 1 g of sample). The mixture was boiled to 150 °C for 4 h, and then deionized water was added to a volume of 25 ml. Same steps were followed to prepare a blank digest. Standard solutions for calibration were prepared by diluting a stock solution of 1000 mg/l (Sigma and Aldrich) of each tested element.

For the determination of soluble sugars, mushroom samples were heated in water at 100 °C for 30 min. The sample solution was filtered and 20 µl of filtrate were used then for normal phase extraction using High Performance Liquid Chromatography (HPLC) at 30 °C (normal phase extraction; column NH_2_ column: 250 × 4.5 mm ID, at a flow rate of 1.2 ml min^−1^). Sugars were detected with a refractive index detector (RID) (mobile phase: mixture of polar-non-polar solution, calibration: using a 2 points concentration). Sugar identification was made by comparing with standards prepared from stock solution of sugars to get concentrations approximate to sample. For the different tests, each represented value is the mean of 3 replicates determination ± standard deviation.

### Statistical analysis

One-way ANOVA (analysis of variance) and Duncan’s multiple range tests were applied using the program Statistical Package for Social Sciences “SPSS25”, at a 95% level of confidence.

## Results and discussion

Mushrooms grow on agricultural wastes by secreting enzymes to digest the surrounding foodstuffs and get their nutrients from the substrate^44^. Therefore, their nutritional composition will largely depend on the chemical composition of the growing substrate^7–12^. In early studies, the changes in mushroom nutritional composition were mostly addressed when various nutrient additives were applied at spawning. In the present study, not only the dose of nano-urea, but also its timing of application had varying effects on the mushroom nutritional composition.

### Mushroom composition

There was punctual improvement in the mushroom protein and fiber contents by 0.64% and 0.19% respectively when 3 g kg^−1^ nano-urea was applied at spawning (C_1_t_1_) and by 0.46% and 0.19% respectively when 5 g kg^−1^ nano-urea was applied after the first harvest (C_2_t_2_) (Table 2).

**Table 2:** Composition of *P. ostreatus* mushrooms cultivated on supplemented substrates.

	Fats (% fw)	Fiber (% fw)	Total carbohydrates (% fw)	Total protein (% fw)
C_0_t_0_	0.16 ± 0.02a	2.69 ± 0.25bc	4.36 ± 0.35a	2.92 ± 0.13c
C_1_t_1_	0.23 ± 0.01d	2.88 ± 0.08d	4.84 ± 0.05b	3.56 ± 0.09e
C_1_t_2_	0.22 ± 0.008cd	1.48 ± 0.03a	7.94 ± 0.04e	2.48 ± 0.04a
C_1_t_3_	0.28 ± 0.02e	2.58 ± 0.02b	6.42 ± 0.02d	2.78 ± 0.03b
C_2_t_1_	0.13 ± 0.01a	2.79 ± 0.10cd	4.44 ± 0.22a	3.00 ± 0.14c
C_2_t_2_	0.12 ± 0.01a	3.49 ± 0.01e	8.12 ± 0.02e	3.38 ± 0.03d
C_2_t_3_	0.18 ± 0.08bc	1.60 ± 0.10a	5.46 ± 0.02c	2.95 ± 0.10c
**Sig. level**				
Dose	0.000	0.000	0.002	0.000
Timing	0.001	0.000	0.000	0.000
Dose × timing	0.997	0.000	0.000	0.000

*C*_*1*_ 3 g kg^−1^, *C*_*2*_ 5 g kg^−1^, *t*_*1*_ supplementation at spawning, *t*_*2*_ supplementation after the first harvest, *t*_*3*_ supplementation at spawning and after the first harvest. Values are means; means within the same column followed by the same letters are not significantly different at *P* < 0.05 according to Duncan’s multiple range test.

The nitrogen source is a major factor that affects the fungus’s enzyme production for biodegradation of a certain substrate^45^. Nitrogen can be transported into the fungus’s living cell in the form of amino acids^46^. Substrate proteins are degraded into amino acids by the extracellular enzymes secreted by the fungus mycelium. Amino acids are then assimilated for subsequent utilization for protein synthesis in the mushroom. The substrate composition and the harvest time of mushrooms may affect their protein content^47^. Values of protein content relative to the first timing of product application (at spawning) correspond to mushrooms of the first harvest, and were higher or comparable to control (3.56% and 3.00% in C_1_t_1_ and C_2_t_1_ respectively compared to 2.92% in control). A low product dose applied at spawning (C_1_t_1_) may have enhanced the enzymatic accessibility of amino acids to the fungus, compared to control. Further, mushrooms consume the substrate’s nutrients as long as they grow, thus nutrient depletion from the substrate causes a reduction in mushroom yields with consecutive flushes^48^. Concerning the second timing of product application (after the first harvest), values of proteins and fibers in mushrooms of C_1_t_2_ and C_2_t_2_ correspond to mushrooms harvested at the second flush. At this stage, a large part of nutrients may have been lost from the substrate, therefore a higher product dose may have been critical to reactivate the process of substrate degradation, which may have caused superiority in both components (proteins and fibers) in mushrooms harvested from the substrate treated with 5 g kg^−1^ compared to those produced by the substrate treated by 3 g kg^−1^ (3.49% and 1.48% in C_2_t_2_ and C_1_t_2_, respectively). These assumptions may be further proved by a detailed analysis of the changes in the enzymatic activity at the level of the substrate at the consecutive stages of mushroom production.

Earlier, an increase of mushroom protein content was reported on the same type of substrate (SOS in mixture with wheat straw) supplemented with wheat bran^25^. In the same trend, an increase in protein content, in the range of 0.41–0.83%, was found when the spent oyster substrate was supplemented with 30% sawdust and 20% date palm fibers^49^. The use of conventional urea (0.5% w/w) caused a 33.6% increase in protein content of mushrooms grown on sugarcane bagasse^31^.

Protein is an important constituent of mushrooms dry matter; because of their high protein content, mushrooms rank between meat and vegetables and could provide a solution to the problem of protein meals in developing countries^50–52^. High-fiber food products offer significant health benefits, reducing the incidence of several human diseases^53^.

On the other hand, fat content (Table 2) increased in mushrooms of C_1_t_1_, C_1_t_2_, C_1_t_3_, and C_2_t_3_, by 0.07, 0.06, 0.12, and 0.02%, but decreased in mushrooms of C_2_t_1_ and C_2_t_2_ by 0.03 and 0.04%, respectively. Furthermore, except in C_2_t_1_, the total carbohydrates content increased by a range of 0.48–3.76% in mushrooms following nano-urea treatment, with the most significant improvement recorded C_2_t_2_. Most of the carbohydrates in mushrooms are non-digestible carbohydrates (NDCs) including oligosaccharides such as trehalose and cell wall polysaccharides, such as chitin, β-glucans, and mannans^54^. Chitin and β-glucans are the major cell wall components of mushroom sclerotium^55^. Therefore, changes in carbohydrates content, as affected by nano-urea treatments suggest a direct effect of the product on the mushroom texture and firmness. Carbohydrate is an essential nutrient for human health, and an efficient source of energy for the human body^56^. However, values of carbohydrates, total protein, fat, and crude fiber contents obtained in nano-urea treated cases were lower than those reported earlier on substrates supplemented by various protein-rich additives^57^.

The amount of sugars in Pleurotus spp. greatly depends on the growth substrate^58^. As a white-rot fungus, *P. ostreatus* is able to degrade lignocellulosic biomass of substrate, into soluble sugars, through the action of complex oxidative and hydrolytic enzymatic systems^59^. To decompose the substrate holocellulose (cellulose and hemicellulose), the fungus needs first to degrade lignin through the action of manganese peroxidase (MnP) and laccase, the major oxidative enzymes responsible for lignin oxidation^60^. Glucose is the most abundant form of cellulose. This sugar was the most abundantly found in mushrooms produced in the treatment C_1_t_3_, causing the highest total sugars (Table 3) compared to all treatments, and an improvement of 0.29% compared to control. This finding may be attributed to higher sugar assimilation from this substrate, directly linked to the highest degree of cellulose degradation, and indirectly to highest lignin degradation, compared to other treatments. In fact, results of Table 4 demonstrate that average values of residual lignin, cellulose, and hemicellulose were the lowest in the substrate C_1_t_3_. Moreover, nano-urea contains 0.01% manganese which may have positively affected the activity of MnP enzyme on substrate lignocellulose, mostly following consecutive applications of a low product dose (3 g kg^−1^). Early findings have proven that such a treatment (3 g kg^−1^ applied at spawning) had positively affected mushroom production, reaching an approximate 110% biological efficiency^33^. The same dose (3 g kg^−1^) applied once caused higher values of residual fibers in the substrates, compared to control. Besides, a higher dose of the product (5 g kg^−1^) increased the degradation of the substrate lignocellulose, compared to other nano-urea treatments, only when applied at spawning, which may explain the punctual increase in fructose content in fruits of the treatment C_2_t_1_ (increase by 0.045%) compared to control. But, a double application of the highest product dose caused lower substrate degradation (higher average values of residual fibers), compared to control, maybe because of excess nitrogen, which may suppress the lignilolytic activity of *P. ostreatus*^61^. Overall, the decrease in sugar content of food is advantageous as excessive sugar intake is associated with adverse health conditions, including obesity, metabolic syndrome and inflammatory diseases^62^.

**Table 3:** Sugar composition of *P. ostreatus* mushrooms cultivated on supplemented substrates.

	Total sugars (% fw)	Fructose (% fw)	Glucose (% fw)	Sucrose (% fw)
C_0_t_0_	0.18 ± 0.02c	0.005 ± 0.000a	0.17 ± 0.02d	0.01 ± 0.007b
C_1_t_1_	0.07 ± 0.01b	0.005 ± 0.000a	0.07 ± 0.000b	0.005 ± 0.000a
C_1_t_2_	0.02 ± 0.000a	0.005 ± 0.000a	0.01 ± 0.000a	0.005 ± 0.000a
C_1_t_3_	0.47 ± 0.008d	0.009 ± 0.001a	0.46 ± 0.02e	0.005 ± 0.000a
C_2_t_1_	0.07 ± 0.008b	0.05 ± 0.007b	0.02 ± 0.001a	0.005 ± 0.000a
C_2_t_2_	0.01 ± 0.000a	0.005 ± 0.000a	0.09 ± 0.01c	0.005 ± 0.000a
C_2_t_3_	0.07 ± 0.01b	0.005 ± 0.000a	0.06 ± 0.000b	0.005 ± 0.000a
**Sig. level**				
Dose	0.000	0.000	0.000	0.201
Timing	0.000	0.000	0.000	0.199
Dose × timing	0.000	0.000	0.000	0.199

*C*_*1*_ 3 g kg^−1^, *C*_*2*_ g kg^−1^, *t*_*1*_ supplementation at spawning, *t*_*2*_ supplementation after the first harvest, *t*_*3*_ supplementation at spawning and after the first harvest. Values are means; means within the same column followed by the same letters are not significantly different at *P* < 0.05 according to Duncan’s multiple range test.

**Table 4:** Averages of hemicellulose, cellulose, and lignin contents in the residual substrates.

	Hemicellulose (% dw)	Cellulose (% dw)	Lignin (% dw)
C_0_t_0_	0.98	23.33	4.46
C_1_t_1_	4.11	23.75	5.83
C_1_t_2_	10.98	25.98	4.5
C_1_t_3_	0.87	16.62	3.04
C_2_t_1_	1.2	23.12	3.9
C_2_t_2_	4.72	24.22	5.74
C_2_t_3_	6.27	26.55	6.28

### Essential amino acids

Nutritionally, essential amino acids are defined as those amino acids that normally are insufficiently synthesized by the organism relative to its needs for maintenance, growth, development, and health, and which must be provided in the diet to meet requirements^63^. They are signaling molecules and gene expression modulators^64^. At the first timing of application (at spawning), it seems that a low dose of nano-urea (3 g kg^−1^) was enough to enhance the assimilation of amino acids from the substrate, while the highest product dose (5 g kg^−1^) has negatively affected it. In fact, the content of all essential amino acids was significantly improved in C_1_t_1_ compared to C_2_t_1_ and control (Table 5). Among all treatments, essential amino acids were the highest in mushrooms of the treatment C_1_t_1_, where threonine, valine, isoleucine, leucine, phenylalanine, histidine, lysine, and methionine contents increased by 0.31, 0.10, 0.05, 0.21, 0.18, 2.05, 0.27, and 0.09%, respectively compared to control. Another nano-supplement, rich in amino acids (Lithovit-Amino25) improved most of the essential amino acids in *P. ostreatus* mushroom when applied at spawning^65^.

**Table 5:** Essential amino acids (%dw) of *P. ostreatus* mushrooms cultivated on supplemented substrates.

	Thr	Val	Ile	Leu	Phe	His	Lys	Met
C_0_t_0_	0.96 ± 0.03b	0.86 ± 0.07c	0.66 ± 0.04d	1.11 ± 0.03d	0.68 ± 0.13bc	2.77 ± 0.03a	0.91 ± 0.07d	0.27 ± 0.01c
C_1_t_1_	1.27 ± 0.01e	0.96 ± 0.13d	0.71 ± 0.00e	1.32 ± 0.01e	0.86 ± 0.01d	4.82 ± 0.03g	1.18 ± 0.01e	0.36 ± 0.01e
C_1_t_2_	0.94 ± 0.05b	0.73 ± 0.03b	0.57 ± 0.02b	0.96 ± 0.02a	0.64 ± 0.02b	3.88 ± 0.08d	0.74 ± 0.02a	0.29 ± 0.01d
C_1_t_3_	1.08 ± 0.02d	0.83 ± 0.02c	0.65 ± 0.02d	1.02 ± 0.02b	0.75 ± 0.02c	3.45 ± 0.01b	0.89 ± 0.03d	0.23 ± 0.02a
C_2_t_1_	0.84 ± 0.04a	0.68 ± 0.04a	0.49 ± 0.01a	0.95 ± 0.02a	0.56 ± 0.05a	4.4 ± 0.00f.	0.84 ± 0.005bc	0.24 ± 0.01a
C_2_t_2_	1.00 ± 0.01c	0.81 ± 0.002c	0.61 ± 0.00c	1.08 ± 0.00c	0.66 ± 0.00b	4.14 ± 0.00e	0.82 ± 0.02b	0.26 ± 0.03b
C_2_t_3_	1.10 ± 0.03d	0.73 ± 0.02b	0.55 ± 0.02b	1.00 ± 0.02b	0.65 ± 0.02b	3.65 ± 0.01c	0.87 ± 0.01cd	0.23 ± 0.01a
**Sig. level**								
Dose	0.000	0.000	0.000	0.000	0.000	0.269	0.000	0.000
Timing	0.000	0.005	0.236	0.000	0.048	0.000	0.000	0.000
Dose × timing	0.000	0.000	0.000	0.000	0.000	0.000	0.000	0.000

*C*_*1*_ 3 g kg^−1^, *C*_*2*_ 5 g kg^−1^, *t*_*1*_ supplementation at spawning, *t*_*2*_ supplementation after the first harvest, *t*_*3*_ supplementation at spawning and after the first harvest, *Thr* threonine, *Val* valine, *Ile* isoleucine, *Leu* leucine, *Phe* phenylalanine, *His* histidine, *Lys* lysine, *Met* methionine, *do* dose, *tim* timing. Values are means; means within the same column followed by the same letters are not significantly different at *P* < 0.05 according to Duncan’s multiple range test.

On the other hand, at the second timing of application (after the first harvest), both product doses have caused significant reduction in essential amino acids contents in mushrooms, compared to control, with few exceptions (respective increase of histidine content by 1.11 and 1.37% in C_1_t_2_ and C_2_t_2_, and of methionine content by 0.02% in C_1_t_2_). At this timing of application, essential amino acids were significantly higher in mushrooms of C_2_t_2_ than C_1_t_2_, except phenylalanine and methionine.

Furthermore, the product application twice during the production cycle (timing 3) caused a significant reduction in the majority of essential amino acids compared to control, with few exceptions; threonine and histidine contents increased by 0.12 and 0.14% in C_1_t_3_ and C_2_t_3_, respectively, while phenylalanine was comparable to control in both treatments. These findings suggest that the assimilation of these three amino acids (threonine, histidine, and phenylalanine) from the substrate is not counteracted by high nitrogen doses accumulating in the growing substrate. Instead, their assimilation mechanism may have required a stronger mycelia growth, thus higher enzyme secretion by the mushroom to degrade the substrate proteins. Overall, the most dominant essential amino acid was histidine, followed by leucine and threonine.

### Non-essential amino acids

Non-essential amino acids have a major role in regulating gene expression, cell signaling pathways, digestion and absorption of dietary nutrients^63^. At the first timing of nano-urea application (at spawning), and similarly to the pattern observed for essential amino acids, the lowest product dose was enough to enhance the assimilation of all non-essential amino acids from the substrate, causing significant improvement of their contents in mushrooms compared to control (Table 6). In C_1_t_1_, contents of aspartic acid, serine, glutamic acid, proline, glycine, alanine, arginine, and cysteine increased by 0.67, 0.43, 0.95, 0.31, 0.22, 0.07, 0.89, and 0.06% compared to control. A higher product dose applied at this timing (C_2_t_1_) caused significantly lower values (for aspartic acid, serine, glutamic acid, glycine, alanine, and arginine), or values comparable to control (proline and cysteine). The higher content of both essential and non-essential amino acids caused higher protein content in mushrooms of C_1_t_1_ compared to C_2_t_1_.

**Table 6:** Non-essential amino acids (% dw) of *P. ostreatus* mushrooms cultivated on supplemented substrates.

	Asp	Ser	Glu	Pro	Gly	Ala	Arg	Cys
C_0_t_0_	1.7 ± 0.16b	0.85 ± 0.04b	4.21 ± 0.21b	0.66 ± 0.01a	0.82 ± 0.12c	1.69 ± 0.10bc	1.59 ± 0.07f	0.15 ± 0.01a
C_1_t_1_	2.37 ± 0.01e	1.28 ± 0.02e	5.16 ± 0.01d	0.97 ± 0.04c	1.04 ± 0.01d	1.76 ± 0.01d	2.48 ± 0.01g	0.21 ± 0.004e
C_1_t_2_	1.84 ± 0.04cd	0.94 ± 0.04c	3.91 ± 0.05a	0.68 ± 0.05a	0.73 ± 0.05ab	1.63 ± 0.02ab	1.26 ± 0.02a	0.16 ± 0.01ab
C_1_t_3_	1.77 ± 0.02bc	0.99 ± 0.01d	4.7 ± 0.10c	0.7 ± 0.10ab	0.78 ± 0.02bc	1.86 ± 0.02e	1.52 ± 0.05e	0.19 ± 0.02d
C_2_t_1_	1.54 ± 0.04a	0.81 ± 0.01a	3.86 ± 0.05a	0.65 ± 0.01a	0.7 ± 0.003a	1.59 ± 0.07a	1.42 ± 0.002c	0.15 ± 0.004a
C_2_t_2_	1.87 ± 0.004d	0.97 ± 0.003cd	4.71 ± 0.003c	0.76 ± 0.02b	0.84 ± 0.003c	2.37 ± 0.002f.	1.32 ± 0.002b	0.17 ± 0.002bc
C_2_t_3_	1.78 ± 0.02bcd	0.95 ± 0.02c	4.72 ± 0.004c	0.65 ± 0.02a	0.72 ± 0.002ab	1.75 ± 0.02cd	1.47 ± 0.004d	0.18 ± 0.01cd
**Sig. level**								
Dose	0.000	0.000	0.000	0.000	0.000	0.000	0.000	0.000
Timing	0.000	0.000	0.000	0.000	0.000	0.000	0.000	0.002
Dose × timing	0.000	0.000	0.000	0.000	0.000	0.000	0.000	0.000

*C*_*1*_ 3 g kg^−1^, *C*_*2*_ 5 g kg^−1^, *t*_*1*_ supplementation at spawning, *t*_*2*_ supplementation after the first harvest, *t*_*3*_ supplementation at spawning and after the first harvest, *Asp* Aspartic acid, *Ser* serine, *Glu* GLUTAMIC acid, *Pro* proline, *Gly* glycine, *Ala* Alanine, *Arg* arginine, *Cyst* cysteine, *do* dose, *tim* timing. Values are means; means within the same column followed by the same letters are not significantly different at *P* < 0.05 according to Duncan’s multiple range test.

On the other hand, when nano-urea was applied at timing 2 (after the first harvest), a higher product dose was essential to ameliorate the assimilation of most non-essential amino acids (except arginine) from the substrate, improving their contents by a range of 0.02–0.68% in mushrooms of C_2_t_2_ compared to control.

Improvement in protein content in mushrooms of C_2_t_2_ compared to C_1_t_2_ is explained by higher contents of most essential and all non-essential amino acids in the former compared to the latter treatment. Besides, superior essential and non-essential amino acids contents caused the highest protein content in mushrooms of C_1_t_1_ compared to all tested treatments.

In contrast to the pattern observed for essential amino acids, the double application of nano-urea (timing 3) may have ameliorated the assimilation of most non-essential amino acids from the substrate, causing significant improvement of their contents in mushrooms. However, the double product application had negatively affected the mushroom content in glycine and arginine, independently from the dose applied, and in aspartic acid and proline, following the application of the highest dose. The mechanism of assimilation of these four amino acids may have been counteracted by the progressive accumulation of nitrogen in the substrate. However, a further investigation of the enzymatic activity as affected by nano-urea application may better confirm these findings.

The amino acids of mushrooms produced from nano-urea treated substrates were higher than values of amino acids of early studies^57^. Glutamic acid was the most dominant non-essential amino acids in all treatments, followed by alanine, aspartic acid, and occasionally by arginine. In other reports, asparatic acid, glutamic acid and arginine were detected as three main amino acids in *P. ostreatus* mushrooms^66–68^.

### Minerals and heavy metals

Results in Table 7 showed that nano-urea treatment decreased the mushroom phosphorus content by a range of 0.03–0.13% in comparison with control mushrooms. *P. ostreatus* is a phosphorus rich mushroom, which makes it a good contributor in human nutrition^4^. However, high levels of phosphorus in food inhibit the intake of calcium causing bones weakness, itchy skin and bone or joints pain leading to chronic kidney disease-mineral bone disorder^69^. On the other hand, nano-urea application had significantly increased the zinc content of mushrooms in all treatments (by a range of 8.3–43.2 ppm), except in C_1_t_1_ and C_2_t_1_ (reduction by 3.9 and 1.2 ppm, respectively). Mushrooms act as good zinc accumulators^70^, this element is highly associated with protein- and carbohydrate- rich foods^71^. Zinc content in mushrooms of C_1_t_2_, C_1_t_3_, and C_2_t_3_ was higher than the safe limit set by the WHO^72^ (60 ppm); while mushrooms of the remaining treatments had safe zinc contents.

**Table 7:** Mineral composition of *P. ostreatus* mushrooms cultivated on supplemented substrates.

	P (%)	Zn (ppm)	Ca (%)	K (%)	Na (%)	Mn (ppm)	Mg (%)	Fe (ppm)
C_0_t_0_	0.678 ± 0.01e	42.5 ± 9.00c	0.004 ± 0.001d	0.36 ± 0.04c	0.008 ± 0.001d	1.40 ± 0.55a	0.02 ± 0.001a	21.60 ± 0.55d
C_1_t_1_	0.64 ± 0.01d	38.6 ± 1.14a	0.002 ± 0.0001b	0.37 ± 0.05c	0.008 ± 0.0001d	1.000 ± 0.000a	0.01 ± 0.0001a	19.80 ± 0.84c
C_1_t_2_	0.58 ± 0.02b	85.66 ± 0.61g	0.001 ± 0.000a	0.27 ± 0.001ab	0.006 ± 0.0001c	1.000 ± 0.000a	0.01 ± 0.0001a	17.00 ± 2.24b
C_1_t_3_	0.62 ± 0.01c	69.52 ± 0.15f	0.003 ± 0.0001c	0.25 ± 0.0001a	0.004 ± 0.0001b	1.40 ± 0.55a	0.04 ± 0.0006a	17.20 ± 0.84b
C_2_t_1_	0.55 ± 0.02a	41.3 ± 0.10b	0.003 ± 0.001c	0.28 ± 0.03ab	0.006 ± 0.0003c	1.000 ± 0.000a	0.01 ± 0.001a	13.60 ± 1.52a
C_2_t_2_	0.65 ± 0.02d	50.78 ± 0.15d	0.002 ± 0.000b	0.31 ± 0.0001b	0.003 ± 0.0001a	1.000 ± 0.000a	0.02 ± 0.000a	20.00 ± 1.00cd
C_2_t_3_	0.61 ± 0.02c	62.52 ± 0.08e	0.002 ± 0.0001b	0.27 ± 0.0001ab	0.0006 ± 0.0001c	1.000 ± 0.000a	0.01 ± 0.000a	16.80 ± 0.84b
**Sig. level**								
Dose	0.043	0.000	0.004	0.173	0.000	0.223	0.336	0.013
Timing	0.002	0.000	0.000	0.000	0.000	0.229	0.359	0.006
Dose × timing	0.000	0.000	0.000	0.000	0.000	0.229	0.263	0.000

*C*_*1*_ 3 g kg^−1^, *C*_*2*_ 5 g kg^−1^, *t*_*1*_ supplementation at spawning, *t*_*2*_ supplementation after the first harvest, *t*_*3*_ supplementation at spawning and after the first harvest. Values are means; means within the same column followed by the same letters are not significantly different at *P* < 0.05 according to Duncan’s multiple range test.

Moreover, there was a statistically significant reduction in mushroom calcium content, by a range of 0.001–0.003%, in all nano-urea treatments. Likewise, mushroom potassium and sodium contents decreased significantly in mushrooms, except in C_1_t_1_, where both elements were comparable to control mushrooms. In general, in Pleurotus spp. the content of sodium is low, while the potassium content is high and this is beneficial from a nutritional point of view^58^. Low sodium contents in food help in is controlling the blood pressure problems^73^.

Iron content decreased by a range of 4.4–8.0 ppm in all treatments, except in C_2_t_2_.The range of mushroom iron content obtained in all nano-urea treatments (16.8–20.0 ppm) was higher than the safe limit set by the FAO/WHO/CODEX^72^ standard safe limit (15 ppm), except in C_2_t_1_ (13.6 ppm). However, values of iron content obtained following nano-urea application got closer to the standard safe limit, compared to values obtained in control mushrooms. A diet rich in iron helps decreasing the incidence of anemia^74^. On the other hand, nano-urea application did not significantly affect manganese and magnesium contents in mushroom. Manganese content obtained in mushrooms from this study was lower^75,76^ or in the range indicated in early studies^77^ and very far from the toxicity level (400–1000 ppm) indicated by WHO^78^ both in treated and non-treated cases. Manganese is an essential nutrient in human diet; it plays a major role in intracellular activities^79^.

In comparison with mushrooms obtained from control substrate, mushrooms of nano-urea treated substrates had higher nickel (increase by a range of 10.8–14.0 ppm) and lead (increase by a range of 10.1–21.01 ppm) contents. Contrarily, copper content decreased by a range of 3.6–10.7 ppm in all treatments, except in C_1_t_2_ (increase of 5.7 ppm) (Table 8). Copper content in mushrooms produced for this study was lower than the one reported by Gebrelibanos et al.^71^, but in the range stated by Elekes et al.^80^, and in accordance with the FAO/WHO/CODEX^72^ standard safe limit (lower than 40 ppm).

**Table 8:** Heavy metal composition of *P. ostreatus* mushrooms cultivated on supplemented substrates.

	Cu (ppm)	Ni (ppm)	Pb (ppm)
C_0_t_0_	13.9 ± 0.1f.	8.8 ± 0.16a	6.2 ± 0.07a
C_1_t_1_	6.76 ± 0.11b	19.6 ± 0.16bc	16.32 ± 0.08b
C_1_t_2_	19.6 ± 1.14g	22.7 ± 0.10c	27.26 ± 0.11f.
C_1_t_3_	3.18 ± 0.15a	21.66 ± 0.11c	21.62 ± 0.08d
C_2_t_1_	8.4 ± 0.16c	22.3 ± 0.07c	27.18 ± 0.08f
C_2_t_2_	11.26 ± 0.11e	20.8 ± 1.48b	17.58 ± 0.15c
C_2_t_3_	10.26 ± 0.18d	22.8 ± 0.16c	23.16 ± 0.18e
**Sig. level**			
Dose	0.447	0.005	0.000
Timing	0.000	0.000	0.000
Dose × timing	0.000	0.000	0.000

*C*_*1*_ 3 g kg^−1^, *C*_*2*_ 5 g kg^−1^, *t*_*1*_ supplementation at spawning, *t*_*2*_ supplementation after the first harvest, *t*_*3*_: supplementation at spawning and after the first harvest. Values are means; means within the same column followed by the same letters are not significantly different at *P* < 0.05 according to Duncan’s multiple range test.

The activation of some enzyme systems could be induced by trace amounts of nickel, while when in high levels, nickel can lead to serious toxicity^81^. Mushrooms produced in nano-urea treated substrates presented a dramatic increase in nickel content. It was higher than the safe range 0.05–5 ppm reported for plant foods^82^. Mushrooms produced by the non-treated substrate showed a higher percentage of lead compared to that reported earlier (0.04 mg kg^−1^)^83^ and exceeding the permitted level stated by the EU commission^84^ (0.3 mg kg^−1^) and WHO^72^ (2 mg kg^−1^).

Like other mushrooms, Oyster mushroom has the ability to absorb heavy metals from substrates via spacious mycelium^83^. The mineral level of mushrooms is affected by the substrate^85^. Heavy metals bioaccumulation depends on metal concentration in the initial substrate, subtrate pH, cation exchange capacity (CEC), and organic matter content^86,87^. Therefore, different factors may have caused higher accumulation of heavy metals, like Ni, Pb (in all cases), Zn and Fe (in some cases), and lower accumulation of almost all others tested minerals. First, some elements (like Ni, Pb, Fe, Zn, and Cu) were already more abundant in the initial growing substrate. Second, Lithovit-Urea50 is rich in calcium carbonate (33.0%), which may have caused a liming effect to the initially low substrate pH (5.2). Such an effect could have favored the absorption of some minerals over others. It was noted earlier that an excess of lime in the substrate reduces nutrients uptake ability of the fungus^88^, which may explain why a large proportion of minerals (P, Ca, K, Na, Mg, and Mn) were reduced in mushrooms following the product application. Elekes et al.^80^ reported a strong correlation between soil pH and zinc bioaccumulation in wild mushrooms, which may explain why mushroom zinc contents were higher in mushrooms produced from the substrates treated by nano-urea at later stages of the growing process, when the product effect on the substrate pH may have been more influential. Nevertheless, these assumptions require further investigation for the changes in the pH of nano-urea treated substrates during different stages of the growing process. Moreover, the addition of Lithovit-Urea50, containing silicon oxide (SiO_2_), magnesium oxide (MgO), iron (Fe), and manganese (Mn), releasing many cations to the growing medium may have caused a direct effect on the substrate CEC, thus on the mechanism of metal absorption from the substrate, and its subsequent accumulation in mushrooms. One additional factor influencing the absorption of metals in mushrooms is the competition with other metal ions^89^, therefore, the decrease in certain metals in mushrooms of this study, like copper, for example, could be due to the competition caused by nickel, lead, and zinc, the bioaccumulation of which was in general higher under the present experimental conditions.

## Conclusion

Among the different tested timing and doses of nano-urea application, a low product dose (3 g kg^−1^) applied at spawning was enough to enhance many nutritional attributes of *P. ostreatus* mushroom, improving its proteins, essential and non-essential amino acids contents, and lowering its sugar content. Such a treatment resulted in a lower accumulation of zinc and copper in mushrooms compared to all nano-urea treated cases. However, keeping in mind that heavy metals accumulation in food products has many negative effects on human health, and that nano-urea application has generally activated the accumulation of nickel and lead in mushrooms, even with the lowest dose applied, it is recommended to investigate the product effect in lower doses (< 3 g kg^−1^) in future investigations.

## Data Availability

All data generated or analyzed during this study are included in this published article.
